# Population genetics of Southern Hemisphere tope shark (*Galeorhinus galeus*): Intercontinental divergence and constrained gene flow at different geographical scales

**DOI:** 10.1371/journal.pone.0184481

**Published:** 2017-09-07

**Authors:** Aletta E. Bester-van der Merwe, Daphne Bitalo, Juan M. Cuevas, Jennifer Ovenden, Sebastián Hernández, Charlene da Silva, Meaghen McCord, Rouvay Roodt-Wilding

**Affiliations:** 1 Department of Genetics, Stellenbosch University, Stellenbosch, South Africa; 2 Universidad Nacional de La Plata (UNLP), División Zoología Vertebrados, Museo de La Plata, La Plata, Argentina; 3 Molecular Fisheries Laboratory, Queensland Government, St Lucia, Queensland, Australia; 4 Sala de Colecciones Biológicas, Facultad de Ciencias del Mar, Universidad Católica del Norte, Coquimbo, Chile; 5 Molecular Biology Laboratory, Center for International Programs, Veritas University, San José, Costa Rica; 6 Fisheries Research, Department of Agriculture, Forestry and Fisheries, Cape Town, South Africa; 7 South African Shark Conservancy, Old Harbour Museum, Hermanus, South Africa; National Cheng Kung University, TAIWAN

## Abstract

The tope shark (*Galeorhinus galeus* Linnaeus, 1758) is a temperate, coastal hound shark found in the Atlantic and Indo-Pacific oceans. In this study, the population structure of *Galeorhinus galeus* was determined across the entire Southern Hemisphere, where the species is heavily targeted by commercial fisheries, as well as locally, along the South African coastline. Analysis was conducted on a total of 185 samples using 19 microsatellite markers and a 671 bp fragment of the NADH dehydrogenase subunit 2 (*ND2*) gene. Across the Southern Hemisphere, three geographically distinct clades were recovered, including one from South America (Argentina, Chile), one from Africa (all the South African collections) and an Australia-New Zealand clade. Nuclear data revealed significant population subdivisions (F_ST_ = 0.192 to 0.376, p<0.05) indicating limited gene flow for tope sharks across ocean basins. Marked population connectivity was however evident across the Indian Ocean based on Bayesian clustering analysis. More locally in South Africa, F-statistics and multivariate analysis supported moderate to high gene flow across the Atlantic/Indian Ocean boundary (F_ST_ = 0.035 to 0.044, p<0.05), with exception of samples from Struisbaai and Port Elizabeth which differed significantly from the rest. Discriminant and Bayesian clustering analysis indicated admixture in all sampling populations, decreasing from west to east, corroborating possible restriction to gene flow across regional oceanographic barriers. Mitochondrial sequence data recovered seven haplotypes (*h* = 0.216, π = 0.001) for South Africa, with one major haplotype shared by 87% of the individuals and at least one private haplotype for each sampling location except Port Elizabeth. As with many other coastal shark species with cosmopolitan distribution, this study confirms the lack of both historical dispersal and inter-oceanic gene flow while also implicating contemporary factors such as oceanic currents and thermal fronts to drive local genetic structure of *G*. *galeus* on a smaller spatial scale.

## Introduction

Elasmobranchs are currently regarded as one of the most vulnerable extant vertebrate groups and many of the species are threatened with extinction [1]. The exploitation of elasmobranchs has been steadily increasing raising concerns over the sustainability of this marine resource and the impacts to the marine ecosystem globally [2]. Most elasmobranchs (especially sharks) are vulnerable to fishing pressures due to the relatively *K*-selected traits they exhibit such as low fecundity, late sexual maturity and a long lifespan with slow growth rates [3]. Further, limited baseline data exists for species-specific landings since historically elasmobranchs were of low economic value and a lesser priority in terms of fisheries management. In general, the assessment of the spatial extent of populations has been hampered by a lack of fisheries independent data, species-specific assessments and limited understanding of transoceanic movement patterns [4]. In order to implement regional management strategies for exploited elasmobranchs, information on migration patterns and genetic population structure is needed to monitor the effect of fishing on individual species along a given stretch of coastline [5]. This could lead to a more integrated approach to fisheries management, where species showing different levels of population subdivision over similar spatial scales, should be co-managed [6,7].

The tope shark (*Galeorhinus galeus* Linnaeus 1758) is a commercially important shark species distributed in temperate waters around the world [8]. Tope is harvested for its high value fillets, sold as flake and is one of the most commercially valuable sharks in South Africa [4]. Across the Southern Hemisphere, the species is heavily targeted in demersal shark fisheries and is therefore listed as vulnerable globally by the International Union for the Conservation of Nature (IUCN) [9]. Despite its commercial importance, limited data on landings exist and Tope is often lumped with similar species. In Chilean waters, for example, landings of *G*. *galeus*, *Mustelus mento*, *M*. *whitney* and *Squalus acanthias*, are lumped under the generic local name “tollo” [10,11]. In the south-western Atlantic (SWA), *G*. *galeus* is ranked as critically endangered and was subject to intensive fishing throughout its distribution. Though drastic declines have occurred, the population continues to be fished without restraint in Argentina and Uruguay [12,13,14]; indeed, new access was granted to a large number of artisanal fishers in the late 1990. Declines in tope shark have been most marked in Brazil and Uruguay, where the Catch Per Unit Effort (CPUE) has declined to nearly zero. In Argentina, the CPUE for the trawler fleet has declined by around 80%, attributed to recruitment overfishing during the 1990s [9]. It is believed that *G*. *galeus* comprises only one population extending across Brazil, Uruguay and Argentina with large aggregations of sharks moving in to closed bays of northern Argentina during spring for parturition [15]. *Galeorhinus galeus* also has an Indo-South Pacific distribution in the Southern Hemisphere wherein it occupies the temperate waters of Australia and New Zealand [9]. In Australia, the species is landed primarily in southern waters, including Tasmania, and is considered overfished and is afforded protected species status [9,16,17]. The species occurs throughout New Zealand’s entire exclusive economic zone (EZZ) where it is considered a sustainable fishery. The New Zealand fisheries mandated numerous restrictions on the commercial harvesting of *G*. *galeus* and as of 1986, implemented eight quota management areas (QMAs). Despite this and genetic evidence for one panmictic population [18], *G*. *galeus* in Australia and New Zealand is currently managed as separate stocks [19].

In South Africa, the commercial fishery for *G*. *galeus* has existed since the 1930s with major landing sites occuring off the south-west coast at Saldanha Bay, Cape Town, Hout Bay, Gans Bay and Struisbaai [20]. Heavy unmanaged fishing resulted in a decline in catches by the 1940s, catches have not returned to pre–World War II levels [21]. The species is listed as vulnerable in South Africa and is threatened by over-exploitation while management is made difficult by a lack of species-specific catch data and non-cohesive fishing regulations across different coastal management zones [21,22]. With the exception of preliminary population genetic data [23], very little information exists for the migratory patterns of this species in South African waters.

The marine realm is a dynamic environment with fluctuating ocean currents and temperatures, all of which can act as drivers of specific dispersal patterns and hence population structure. Despite high dispersal abilities for some coastal sharks, several studies have confirmed different levels of population genetic subdivision over various spatial scales [6,18,24,25]. There is increasing evidence of deep divergences between ocean basin lineages related to paleoceanographic changes, including closure of corridors such as evidenced by the Tethys Seaway [26,27,28]. Futhermore, genetic breaks may be shaped by known biogeographic barriers including ocean currents and temperatures. Certain life history traits, such as philopatric behaviour, have also explained population structure observed in coastal species [29,30]. There are a number of traditionally recognised biogeographic barriers across the Southern Hemisphere: most notably the Eastern South Pacific Barrier (EPB), the Mid-Atlantic Barrier (MAB) and the Benguela Barrier (BB) [31]. The EPB and MAB encompass over 5000km and 2800km of oceanic expanse respectively and have resulted in the complete isolation of populations of coastal species associated with continental shelves [27,32,33,34]. Conversely, these barriers show no to little effect in pelagic species that are highly vagile [26,35,36,37]. Around southern Africa, the BB across the southern tip of Africa, resulting from the cold-water upwelling of the Benguela Current has been reported to restrict gene flow between southern Atlantic and southern Indian Ocean populations of tropical and subtropical sharks such as *Sphyrna lewini* [26] and *M*. *mustelus* [7]. In addition, thermal barriers created by contrasting oceanic currents such as the sharp transition zone along the SWA where the warm Brazil current from the north meets the cold Malvinas current from the south impact gene flow in coastal sharks [38,39].

Previous population genetic studies have supported distinct continental populations of *G*. *galeus*, which are structured along a latitudinal gradient. These studies suggested *G*. *galeus* to have a strong affinity for cool temperate waters limiting their ability to cross warm temperate waters [18,33]. However, none of these studies resolved the genetic connectivity of *G*. *galeus* across all the known barriers and transition zones of the Southern Hemisphere. Here, patterns of gene flow were assessed between geographic samples separated by apparent regions of unsuitable environmental conditions along the species’ range based on the following hypotheses: (1) genetic discontinuity exists across the Southern Hemisphere oceans including the south Pacific, south Atlantic and south Indian Oceans and (2) genetic discontinuity exists across the Indian/Atlantic Ocean boundary with differentiation found between Atlantic and Indian *G*. *galeus*. A dual-marker approach was applied where variation in the mitochondrial *ND2* gene and 19 microsatellite markers were used to assess genetic diversity and population connectivity of *G*. *galeus* across the Southern Hemisphere and the South African coastline.

## Material and methods

### Sample acquisition and DNA extraction

**Fig 1:**
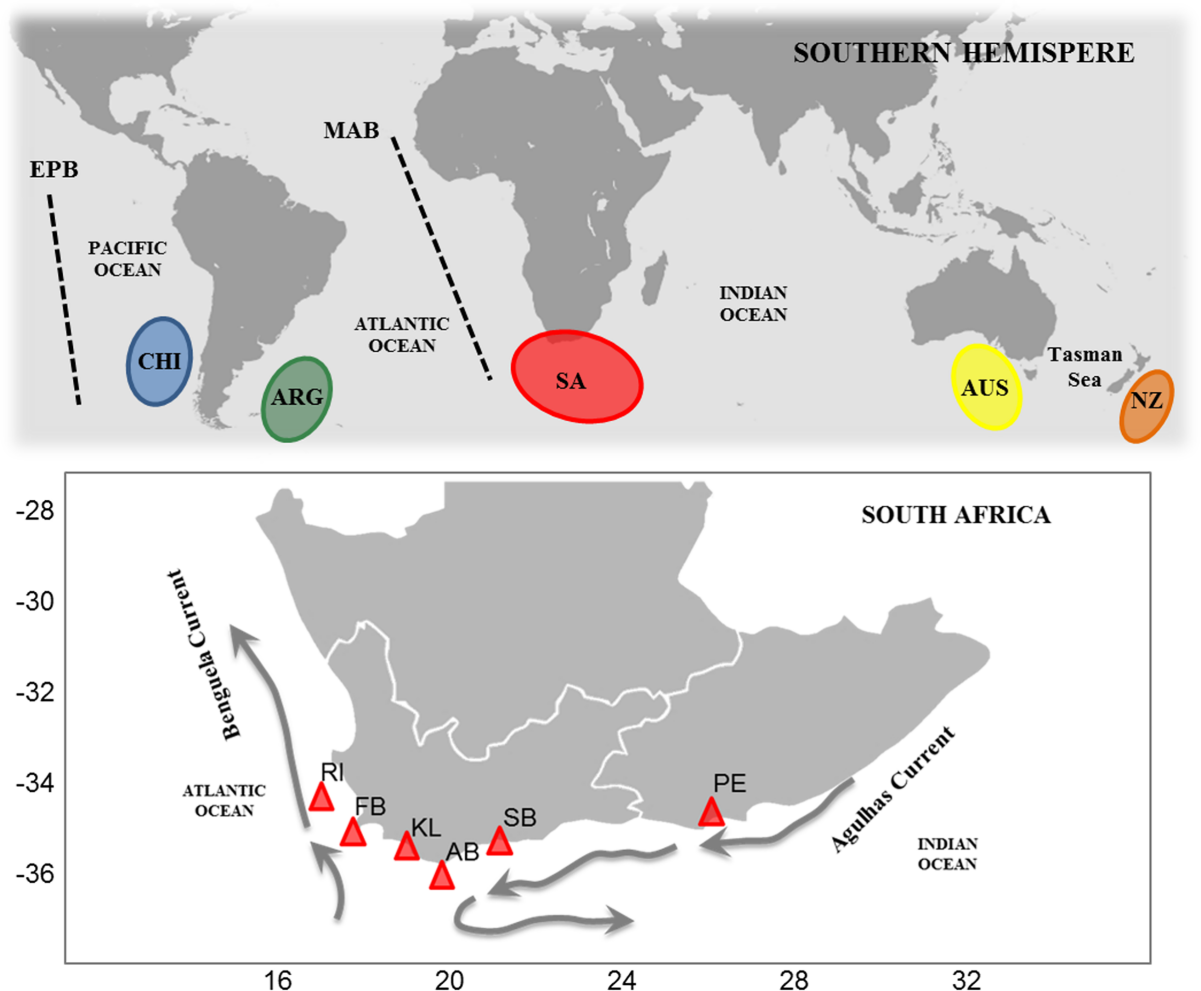
Sampling locations of *Galeorhinus galeus*. Map showing the major biogeographic barriers and oceanic currents across the Southern Hemisphere and South Africa. The main biogeographic barriers indicated by the dashed lines are the Eastern South Pacific Barrier (EPB) and the Mid-Atlantic Barrier (MAB). Sampling codes: Chile (CHI), Argentina (ARG), South Africa (SA), Australia (AUS), New Zealand (NZ); Robben Island (RI), False Bay (FB), Kleinmond (K), Agulhas Bank (AB), Struisbaai (SB) and Port Elizabeth (PE).

Across the Southern Hemisphere, 185 fin clips or muscle biopsies were collected including 22 from Chile, 10 from Argentina, 124 from South Africa, nine from Australia and 20 from New Zealand (Fig 1, S1 Table). Genetic samples collected specifically for this study included those from Argentina, Australia and South Africa. The samples from Chile and New Zealand were acquired in the course of other research and were also included in a previous study on tope [18]. In Argentina, sampling was carried out in compliance with the fishery act # 217/07 for sustainable fishing of coastal sharks in the Province of Buenos Aires. Sharks were all captured and released inside the Bahía San Blás Marine Protected Area by anglers of the Conservar Tiburones en Argentina project. The Australian samples were collected under Department of Primary Industries Parks Water and Environment permit # 11055 and with approval from the University of Tasmania Animal Ethics Committee (# A0011882). More locally, the South African samples were collected by research and commercial vessels according to protocols and permits (# RES2012/59) approved by the Department of Agriculture, Fisheries and Forestry (DAFF), South Africa and covered most of the species’ South African range over which exploitation occurs. Fin clips collected by DAFF scientists were taken from the trailing edge of the second dorsal fin using small surgical scissors and sharks were released with efforts taken to minimize stress and mortality. The majority of the sharks were mature adults (>100cm) and collected between May and September 2012. All sampling populations were mixed-sex. The samples from Struisbaai were collected from dead animals already captured by a commercial fishing company. All genetic samples were handeled according to guidelines of the Research Ethics Committee for Animal Care and Use at Stellenbosch University for work involving tissue samples and not live animals. This included 26 samples from Robben Island (RI), 11 from False Bay (FB), 39 from Kleinmond (KL), ten from Agulhas Bank (AB), 28 from Struisbaai (SB) and ten from Port Elizabeth (PE). All samples except those from Struisbaai originated from fishery observer programs operated by DAFF and some of these samples were included in a previous study on *G*. *galeus* [23]. Genomic DNA was extracted from fin clips or tissue samples using a modified CTAB extraction method with minor modifications [40].

### Laboratory procedures

#### Mitochondrial DNA sequencing

Sequences of the mitochondrial gene NADH dehydrogenase subunit 2 (*ND2*) were analysed for a total of 81 samples of *G*. *galeus* using the species-specific primers of Farrell et al., 2009 [41]. Southern Hemisphere (SH) samples included Chile (6), Argentina (10), South Africa (53), Australia (9) and New Zealand (3) while South African (SA) samples included Robben Island (11), False Bay (7), Kleinmond (12), Agulhas Bank (5), Struisbaai (13) and Port Elizabeth (5). PCR was performed in a 20 μl total volume containing 100 ng template DNA, 1X GoTaq buffer (Anatech, South Africa), 200 μM dNTPs, 0.4 μM of each primer (Integrated DNA Technologies, IDT, South Africa), 2 mM MgCl_2_ (Promega, Wisconsin, USA) and 0.5 U GoTaq DNA polymerase (Anatech, South Africa). PCR amplifications were performed in an Applied Biosystems (ABI) (Life Technologies, California USA) thermal cycler version 2.09 using cycling conditions as described by [41]. Amplicons were sequenced bi-directionally using the BigDye^®^ Terminator 3.1 Cycle Sequencing Kit (Life Technologies, California USA) and a ABI 3730xl Genetic Analyser. All mtDNA sequences were manually edited and aligned using the MUSCLE alignment algorithm available in MEGA 6 [42]. Aligned sequences were trimmed to 599 bp and exported to DNASP 5.10.01 [43] for further analysis.

#### Microsatellite genotyping

A total of 185 *G*. *galeus* individuals were genotyped using ten species-specific microsatellites developed by Chabot et al., 2011 [44] and nine cross-species markers previously developed for *Mustelus henlei* and *M*. *canis* [45,46]. Southern Hemisphere samples included Chile (22), Argentina (10), South Africa (124), Australia (9) and New Zealand (20). South African samples included Robben Island (26), False Bay (11), Kleinmond (39), Agulhas Bank (10), Struisbaai (28) and Port Elizabeth (10). Three multiplex PCRs were conducted based on primer pair combinations and multiplex panels previously optimised for use in *G*. *galeus* [23]. The PCR cycling profile recommended in the Qiagen Multiplex kit user’s manual was used. Subsequent to capillary electrophoresis, microsatellite allele sizes were scored manually using the LIZ^®^ 600 internal size standard and GeneMapper^®^ 4.0 software (ABI, Life Technologies, California, USA). Particular care was taken with allele scoring and control samples were added with each independent capillary electrophoresis run.

### Data analysis

#### Mitochondrial data

The software DNASP and ARLEQUIN 3.5 [47] were used to calculate molecular diversity indices such as the number of segregating sites (*K*), number of haplotypes (*H*), haplotype diversity (*h*) and nucleotide diversity (π). Genetic structure across sampling sites was investigated using two different approaches. Firstly, an analysis of molecular variance (AMOVA) [48] was conducted in ARLEQUIN using 1,000 permutations to determine the variance components and fixation indices (Ф-statistics) at a single level followed by testing hierarchical subdivision between the three Southern Hemisphere oceans: among groups (Ф_CT_), among populations (Ф_SC_), and within populations (Ф_SC_). The Kimura-2 (K2) model selected according to the Bayesian Information Criterion (BIC) generated in MEGA [42] was employed for both the Southern Hemisphere (regional) and South African (local) datasets. Secondly, pairwise genetic differences (Φ_ST_) based on haplotype frequencies were estimated across the Atlantic, South Indian and South Pacific oceans. Pairwise Φ_ST_ values were computed in ARLEQUIN using 20,000 permutations for both Southern Hemisphere and South African datasets. Sequential false discovery rate (FDR) corrections of the significant values were performed following the Benjamini and Hochberg (B–H) method [49]. The reconstruction of genealogies was performed using phylogenetic algorithms in order to estimate the relationship between haplotypes without ambiguities or unresolved connection [50]. A phylogenetic tree of the mtDNA sequences was estimated using a maximum likelihood (ML) approach in PHYML 3.0 [51] based on the Kimura-2 (K2) model. For tree searching and level of branch support, default settings were used. The ML tree was imported into HAPLOVIEWER [50] to create a haplotype network.

To assess the demographic history of the populations, past demographic and population expansions were evaluated using two methods. Firstly, using the neutrality test, computation of Tajima’s *D* [52] and Fu’s F_S_ [53] statistics and significance values were tested by 20,000 coalescent simulations (significance at α ≤ 0.05) under the infinite-sites model in ARLEQUIN. Secondly, nucleotide mismatch distributions of the pairwise differences were obtained for each sampling population (20,000 permutations). The observed distribution was assessed against models of constant population size and population growth-decline to corroborate the significance between observed and expected mismatch distribution patterns using DnaSP [43]. In addition, corresponding Harpending’s raggedness (H_R_) and sum of squared deviations (SSD) indices [54] were calculated in ARLEQUIN to determine whether any observed mismatch distributions were drawn from an expanded population (small values) or a stationary one (large values).

#### Microsatellite data

Departure from the expectations of Hardy-Weinberg Equilibrium (HWE) was examined locus by locus and across each geographic sample in ARLEQUIN. Linkage disequilibrium (LD) between all pairs of loci was also tested in ARLEQUIN, followed by correction for multiple comparisons. Microsatellite scoring errors due to stuttering, large allele dropouts and null alleles were assessed in MICROCHECKER 2.2.3 [55]. Indices of genetic diversity such as mean number of alleles (NA), the effective number of alleles (NE), unbiased expected heterozygosity (uHE) and inbreeding coefficient (FIS) were estimated for each sampling population in GENALEX 6.5 [56]. Given the uneven sample sizes, rarefied private allelic richness (*П*_*s*_) was computed in HP-RARE 1.1 [57] using the rarefaction method with a minimum sample size of n  =  20 gene copies. To test for genetic homogeneity across the Southern Hemisphere and South Africa, a single level AMOVA was conducted in ARLEQUIN for both datasets. In addition, AMOVA was conducted to test for genetic subdivision across the three ocean basins and within South Africa testing *a priori* defined grouping of Atlantic- (RI, FB, KL, AB) versus South Indian Ocean sampling populations (SB, PE). The genetic distance matrix for all AMOVAs was estimated by pairwise differences and the significance levels of the variance components and F-statistics values were tested by 20,000 nonparametric permutations. Pairwise F_ST_ was estimated for all pair of samples and significance was tested using 20,000 permutations in ARLEQUIN. A false discovery rate was determined for multiple tests using the B–H method and applied to minimise type I errors. Tests for isolation-by-distance (IBD) were performed for the South African samples using the web interface isolation by distance web service (IBDWS) 3.23 [58] by plotting linearized F_ST_ values against corresponding minimum geographical distances. Geographic distances were measured with the path tool option in GoogleEarth 6.2.2 (Google Inc.) using the shortest path, via sea, between any two sampling locations and assuming that *G*. *galeus* travels along the coast. Significance was tested by 30,000 randomisations of the data.

Discriminant analysis of principal components (DAPC), a non-model-based (multivariate) clustering method, was implemented in the R package ADEGENET [59]. The DAPC analysis was used here for visual representation of genotypic partitioning of Southern Hemisphere and South African populations, respectively. First, the *find*.*clusters* function, which runs successive *K*-means clustering with increasing number of clusters (*k*), was used to assess the number of clusters which maximizes between group variance and minimizes within group variance [60]. For selecting the optimal *k*, we applied the Bayesian Information Criterion (BIC) for assessing the best supported model. Then, DAPC was performed on the pre-defined clusters based on geographical sampling location (i.e. *k* = *K*) using the *dapc* function. Finally, a Bayesian clustering analysis was performed in STRUCTURE 2.3.4 [61] to detect the most likely number of ancestral genetic clusters (K). Fifteen iterations were run for each expected cluster setting K from 1 to 6 for the regional dataset and 1 to 7 for the local dataset. Markov chain Monte Carlo (MCMC) simulation runs of 10^6^ iterations were made with 10^5^ burn-in periods using an admixture model with correlated allele frequencies. The web-based STRUCTURE HARVESTER 0.6.93 software [62] was used to determine the number of K first by plotting the mean log probability of each successive K and then using the Delta K method following Evanno et al., 2005 [63]. The program CLUMPAK [64] was used for the graphical representation of the STRUCTURE results. As the Evanno method of each K revealed hierarchical structure of the regional data set (K = 2), STRUCTURE was rerun separately on each of the main identified clusters which were the South Pacific cluster (Group 1 = CHI + NZ) and the Indo-Atlantic cluster (Group 2 = ARG + SA + AUS). For the South African dataset, simulations were also run with prior information on sampling location and applying the non-admixture model.

## Results

### Mitochondrial and nuclear descriptive statistics of *G*. *galeus*

#### Regional (Southern Hemisphere)

**Fig 2:**
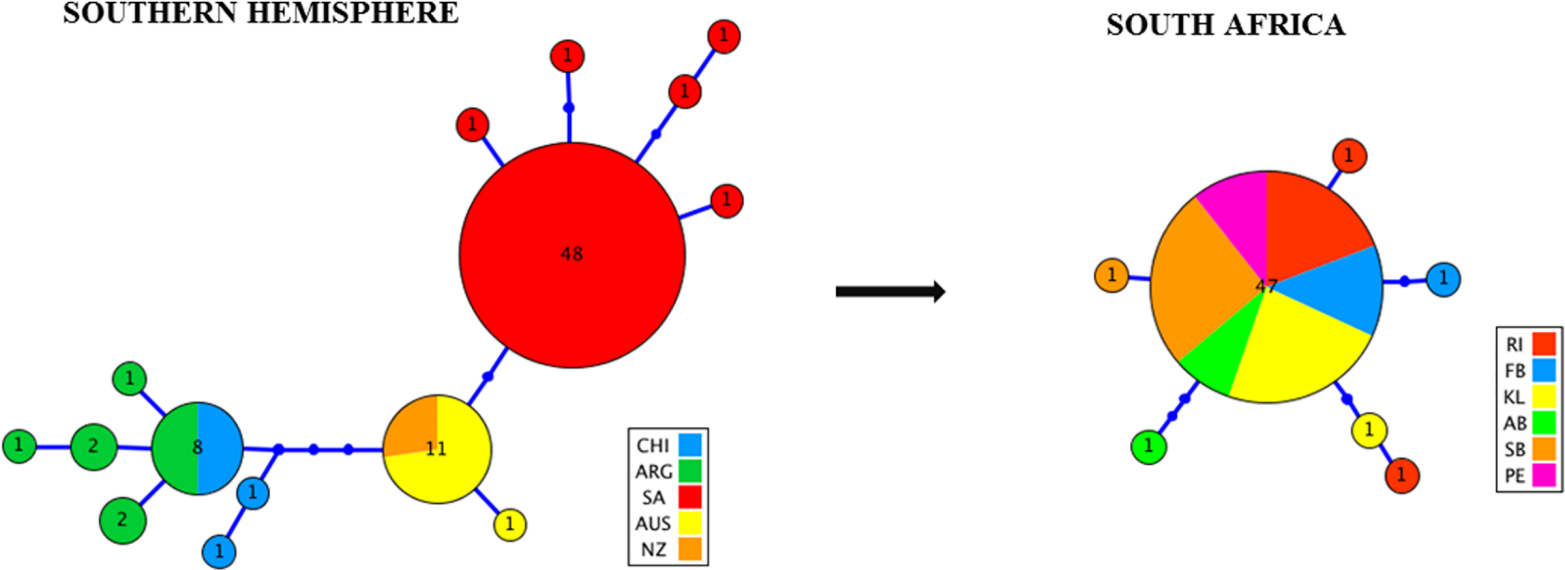
Global and local haplotype genealogy of *Galeorhinus galeus* based on a maximum likelihood tree of *ND2*. Circles represent the haplotypes with area being equivalent to frequency. Each line indicates one mutational step between haplotypes and small dark blue circles indicate hypothetical missing haplotypes.

A 599 bp fragment of the *ND2* gene was sequenced and analysed for a total of 96 *G*. *galeus* samples. Across the Southern Hemisphere, this resulted in a total of 15 haplotypes ranging from one (NZ) to six (SA) per geographical location. The overall haplotype diversity (*h*) was 0.626 ± 0.057 with a nucleotide diversity (π) of 0.004 ± 0.001 (Table 1). A single haplotype was shared between Chile and Argentinia and another one between New Zealand and Australia, while all other haplotypes were unique to their geographical locations. No haplotypes were shared between Argentina and South Africa and therefore across the Atlantic Ocean. The Atlantic Ocean collections (ARG) also showed the highest haplotype diversity (*h* = 0.822 ± 0.097). The haplotype network indicated that haplotypes were almost exclusively associated with one of three *ND2* lineages linked to geographical origin, one including all South African samples, one including all Australian and New Zealand samples and a lineage of South American origin (Fig 2).

**Table 1:** Genetic diversity estimates for all Southern Hemisphere sampling populations of *Galeorhinus galeus*.

Location	Mitochondrial DNA						Microsatellites					
	N	H	H_P_	K	h (± s.d.)	π (± s.d.)	N	N_A_	N_E_	uH_E_	*Пs*	F_IS_
**CHI**	6	3	2	3	0.600 ± 0.215	0.002 ± 0.001	22	10	6.04	0.807	2.66	0.166
**ARG**	10	5	4	4	0.822 ± 0.097	0.002 ± 0.001	10	3	1.95	0.373	0.39	0.040
**SA**	53	7	6	6	0.181 ± 0.071	0.001 ± 0.001	124	11	3.33	0.681	0.77	0.028
**AUS**	9	2	1	1	0.222 ± 0.166	0.001 ± 0.001	9	4	2.55	0.506	0.38	-0.190
**NZ**	3	1	0	0	0.000 ± 0.000	0.000 ± 0.000	20	8	5.52	0.763	1.83	0.349
**All/Avg**	81	18	13	14	0.626 ± 0.057	0.004 ± 0.001	185	36	3.88	0.655	1.21	0.082

For mtDNA *ND2* sequence data: number of samples (N), number of haplotypes (*H*), private haplotypes (H_P_), polymorphic sites (K), haplotype- (h) and nucleotide diversity (π). For microsatellite data: the number of alleles (N_A_), number of effective alleles (N_E_), unbiased expected heterozygosity (uH_E_), rarefied number of private alleles (*Пs*) and inbreeding coefficient (F_IS_).

For the microsatellites, all 19 loci conformed to HWE with the exception of locus *Mca33* and analyses for LD showed that 13 out of 171 locus pairwise comparisons were significant (P<0.002). None of the loci showed evidence of scoring errors due to stuttering, large allele dropouts or the presence of null alleles in MICRO-CHECKER. All diversity estimates for each location are presented in Table 1. Across sampling sites, the total number of alleles (N_A_) and unbiased expected heterozygosity (uH_E_) ranged from 3(ARG) to 11(SA) and 0.373(ARG) to 0.807(CHI) respectively. Unbiased expected heterozygosity (uH_E_) and the effective number of alleles (N_E_) shows nuclear genetic diversity to be higher in the Pacific Ocean (NZ, CHI) relative to the rest of the Southern Hemisphere locations. Mean rarefied private allelic richness per locus and per location averaged 1.21 (Table 1).

#### Local (South Africa)

A total of 53 mtDNA *ND2* sequences from six sampling sites across the coastline of South Africa were analysed. The genetic diversity estimates are summarised in (Table 2). The sequences generated a total of seven haplotypes, with very low levels of haplotype- (*h* = 0.216 ± 0.076) and nucleotide (*π* = 0.001 ± 0.000) diversity overall. One major haplotype was shared amongst 87% of individuals and all sampling sites except Port Elizabeth exhibited at least one unique haplotype (Fig 2). For the microsatellites, all sampling populations were in HWE, with the exception of Agulhas Bank showing significant departure from HWE (*P* < 0.05) at seven (*Mca33*, *McaB39*, *McaB22*, *Gg3*, *Gg11*, *Gg12*, *Gg23*) of the 19 loci. No LD was present for any of the loci pairwise comparisons. MICROCHECKER indicated no scoring errors due to stuttering, large allele dropout or the presence of null alleles. Nuclear genotypic diversity such as unbiased expected heterozygosity and allelic richness were comparable for *G*. *galeus* across the Atlantic and Indian Ocean. The overall number of alleles ranged from N_A_ = 5 to 6 in the Indian Ocean samples, and from N_A_ = 4 to 8 in the Atlantic Ocean samples. Expected heterozygosity was highest for Robben Island (uH_E_ = 0.707) and lowest for Struisbaai (uH_E_ = 0.600) (Table 2).

**Table 2:** Genetic diversity estimates for all South African sampling populations of *Galeorhinus galeus*.

Location		Mitochondrial DNA						Microsatellites				
	N	H	H_P_	K	h (± s.d.)	π (± s.d.)	N	N_A_	N_E_	H_O_	uH_E_	F_IS_
**RI**	11	3	2	3	0.345 ± 0.030	0.001 ± 0.001	26	8	3.686	0.615	0.707	0.141
**FB**	7	2	1	2	0.286 ± 0.196	0.001 ± 0.001	11	5	3.050	0.630	0.641	0.021
**KL**	12	2	1	2	0.167 ± 0.134	0.001 ± 0.001	39	7	3.232	0.692	0.679	-0.025
**AB**	5	2	1	3	0.400 ± 0.237	0.002 ± 0.001	10	4	2.851	0.705	0.615	-0.134
**SB**	13	2	1	1	0.154 ± 0.126	0.002 ± 0.001	28	6	2.726	0.648	0.600	-0.045
**PE**	5	1	1	0	0.000 ± 0.000	0.000 ± 0.000	10	5	3.003	0.786	0.646	-0.210
**All/Avg**	53	12	7	11	0.216 ± 0.076	0.001 ± 0.000	124	35	3.091	0.679	0.669	-0.042

For mtDNA *ND2* sequence data: number of samples (N), number of haplotypes (*H*), private haplotypes (H_P_), polymorphic sites (K), haplotype- (*h*) and nucleotide diversity (π). For microsatellite data: number of alleles (N_A_), number of effective alleles (N_E_), observed heterozygosity (H_O_), unbiased expected heterozygosity (uH_E_), and inbreeding coefficient (F_IS_).

### Population connectivity of *G*. *galeus*

#### Regional (Southern Hemisphere)

Based on the *ND2* gene, genetic differentiation was evident among geographic sampling populations since based on AMOVA, only a small percentage of variation was explained by the within-population level of subdivision while a significant level of variation amongst the geographic populations existed (Ф_ST_ = 0.895, *P* < 0.05). Further grouping hypotheses to test for structuring between ocean basins were not significant (Ф_CT_ = 0.113 to 0.460, *P* > 0.05), irrespective of South Africa being grouped with the Atlantic- or Indian Ocean (Table 3). All of the pairwise comparisons of Ф_ST_ values showed statistically significant differentiation after correcting for multiple tests (Ф_ST_ = 0.151 to 0.934, *P* < 0.05) except for between New Zealand and Australia (Table 4). Overall, this indicated strong inter-continental structure with the highest genetic differentiation found between samples from South Africa and Argentina (Ф_ST_ = 0.934, *P* < 0.05). Substantial population isolation was evident within the Atlantic (ARG, SA), Indian (SA, AUS) and Pacific Ocean (NZ, CHI) samples.

**Table 3:** Analysis of molecular variance (AMOVA) across the Southern Hemisphere of *Galeorhinus galeus* based on mtDNA *ND2* sequence and microsatellite data.

Marker	Hypothesis tested	Source of variation	% variation	Fixation index
**mtDNA**	**Panmixia**	Among locations	87.64	Φ_ST_ = 0.895*
	**Inter-oceanic (SA with Atlantic)**	Within locations	17.68	
		Among groups	11.26	Φ_CT_ = 0.113
		Among locations	58.63	Φ_SC_ = 0.661*
		Within locations	30.11	Φ_ST_ = 0.699*
	**Inter-oceanic (SA with Indian)**	Among groups	64.96	Φ_CT_ = 0.460
		Among locations	22.68	Φ_SC_ = 0.831*
		Within locations	17.68	Φ_ST_ = 0.909*
**Microsatellites**	**Panmixia**	Among locations	13.65	F_ST_ = 0.137
		Within locations	86.35	
	**Inter-oceanic (SA with Atlantic)**	Among groups	5.70	F_CT_ = 0.057
		Among locations	8.88	F_SC_ = 0.094*
		Within locations	85.42	F_ST_ = 0.146*
	**Inter-oceanic (SA with Indian)**	Among groups	8.060	F_CT_ = 0.081
		Among locations	6.810	F_SC_ = 0.074*
		Within locations	85.130	F_ST_ = 0.149*

*Statistically significant at *P* < 0.05

**Table 4:** Pairwise Φ_ST_ values for mtDNA (below diagonial) and pairwise F_ST_ values for microsatellite data (above diagonial) among sampling locations across the Southern Hemisphere (left) and South Africa (right).

	CHI	ARG	SA	AUS	NZ		RI	FB	KL	AB	SB	PE
**CHI**		0.236*	0.136*	0.171*	0.050*	**RI**		0.011	0.000	0.016*	0.030*	0.030*
**ARG**	0.151*		0.138*	0.330*	0.287*	**FB**	0.002		0.017*	0.048*	0.045*	0.023*
**SA**	0.933**	0.934**		0.097*	0.131*	**KL**	-0.053	0.018		0.003	0.028*	0.040*
**AUS**	0.839**	0.844**	0.873**		0.163*	**AB**	0.050	0.023	0.086		0.020*	0.073*
**NZ**	0.770**	0.798**	0.871**	-0.180		**SB**	0.008	0.047	0.003	0.141		0.091*
						**PE**	-0.089	-0.055	-0.093	0.000	-0.096	

**Statistically significant after a false discovery rate (*P* ≤ 0.027)

*Statistically significant at *P* < 0.05

Genetic differentiation across the Southern Hemisphere was further investigated using microsatellite nuclear data. The global AMOVA showed high molecular variation amongst sampling populations (F_CT_ = 0.137, *P* < 0.05) while none of the *a priori* grouping hypotheses tested was significant (Table 3). Similarly, pairwise F_ST_ values indicated high levels of genetic differentiation on an inter-oceanic and intra-oceanic level across the Southern Hemisphere. Pairwise F_ST_ values ranged from 0.050 to 0.330, *P* < 0.05; with the lowest genetic differentiation found between NZ and CHI on opposite sides of the South Pacific Ocean (Table 4).

**Fig 3:**
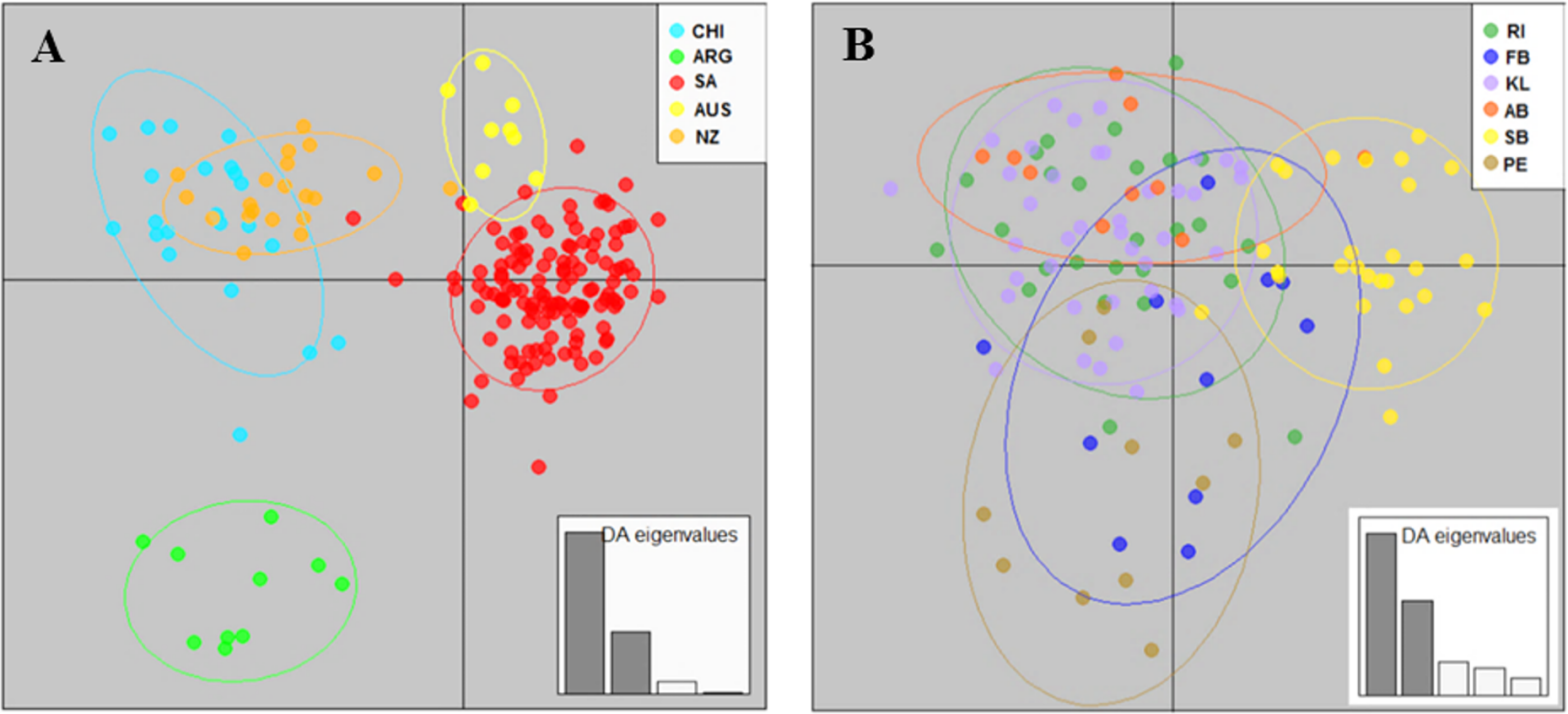
Cluster composition and population differentiation of *Galeorhinus galeus*. Scatterplots generated by the DAPC analysis for sampling populations from (A) the Southern Hemisphere and (B) South Africa respectively.

**Fig 4:**
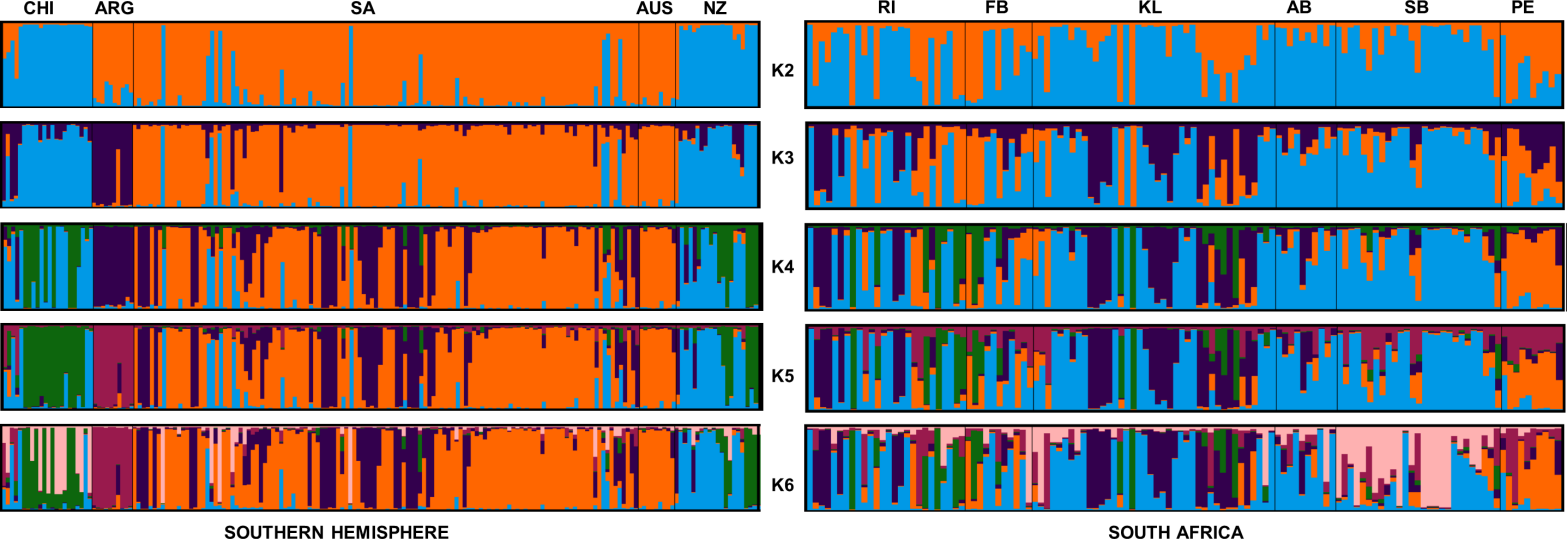
Individual cluster assignments generated from STRUCTURE analysis. This is illustrated by sampling location for K = 2 to K = 6 for both the Southern Hemisphere and the South African sampling populations.

Population structuring was further investigated by ascertaining the relationship between individual genotypes through discriminal analysis of principal components (DAPC). For the K-means method, a k value of nine (the lowest BIC value) represented the best summary of the data that maximized the variance between groups while minimizing the variance within groups. When using the pre-defined clusters based on geographical sampling location, the DAPC plot confirmed strong separation between the five Southern Hemisphere populations of *G*. *galeus* with NZ and CHI as well as SA and AUS showing some overlap (Fig 3). Finally, the true number of populations (K) was investigated using Bayesian clustering analysis. Prior to the application of the Evanno method, the normal distribution of the mean likelihood score (Ln(K)) did not reach a plateau for values of K tested while two clusters (K = 2) were identified and statistically supported based on the Delta K method (S1 Fig). The assignment plot associated with K = 2 implied a strong relationship between the population samples and two genetic groups: 1) CHI + NZ, and 2) ARG, SA and AUS with further structure evident for K > 2 (Fig 4). For this reason, STRUCTURE was run hierarchically for the South Pacific (Group 1 = CHI + NZ) and the Indo-Atlantic clusters (Group 2 = ARG + SA + AUS) respectively. Further subdivision was detected within Group 1 (Delta K was maximum for K = 3) while no further substructure was evident for Group 2 (Delta K was maximum for K = 2) (S2 Fig). The assignment bar plots were investigated for the respective groups and within group 1 assignment was mainly to a single cluster for NZ while shared assignment to three clusters (admixture) was evident in CHI. For group 2, assignment plots indicated almost full membership for ARG and AUS to different clusters with SA showing admixture between the two clusters (S3 Fig).

#### Local (South Africa)

Analysis of the *ND2* sequence variation across six local populations resulted in seven haplotypes, with one common haplotype shared among all the sampling populations. The AMOVA analysis showed no significant molecular variation amongst the sampling populations (Φ_ST_ = 0.013, *P* = 0.255) with most of the variation attributed to amongst individual variation within populations. Also, no significant variation was detected between Indian and Atlantic Ocean samples (Φ_CT_ = -0.018, *P* = 0.752) (Table 5). The pairwise Φ_ST_ values shown in Table 4 corroborated this haplotype genealogy, reflecting high connectivity across the South African populations (*P* > 0.05). For the nuclear data, the single level AMOVA testing for population differentiation, was significant (F_ST_ = 0.025, *P* < 0.05) while the hierarchical AMOVA showed no differentiation between oceanic basins tested for *a priori* (F_CT_ = 0.000, *P* = 0.273) (Table 5). Pairwise F_ST_ values ranged from 0.003 to 0.091, *P* ≤ 0.0363 with the highest pairwise value between the two Indian Ocean populations (PE and SB) (Table 5). Low but significant differentiation between both Struisbaai and Port Elizabeth and the rest of the populations was detected. This could however not be explained by isolation-by-distance as genetic distance was not significantly correlated with geographic distance in South African *G*. *galeus* (R^2^ = 0.238, *P* = 0.1478) (S4 Fig). We therefore continued with tests to detect population genetic structure.

**Table 5:** Analysis of molecular variance (AMOVA) across South Africa of *Galeorhinus galeus* based on mtDNA *ND2* sequence and microsatellite data.

Marker	Hypothesis tested	Source of variation	% variation	Fixation index
**mtDNA**	**Panmixia**	Among locations	1.31	Φ_ST_ = 0.013
		Within locations	98.69	
	**Inter-oceanic (Atlantic vs. Indian)**	Among groups	-1.89	Φ_CT_ = -0.018
		Among locations	2.37	Φ_SC_ = 0.023
		Within locations	99.52	Φ_ST_ = 0.005
**Microsatellites**	**Panmixia**	Among locations	2.48	F_ST_ = 0.025*
		Within locations	97.52	
	**Inter-oceanic (Atlantic vs. Indian)**	Among groups	-0.01	F_CT_ = 0.000
		Among locations	2.48	F_SC_ = 0.025*
		Within locations	97.53	F_ST_ = 0.024*

*Statistically significant at *P* < 0.05

With the DAPC, the sampling population of Struisbaai clustered separately while the Port Elizabeth population also showed less overlap with the rest of the sampling populations (Fig 3). In STRUCTURE using sampling locations as priors, the mean likelihood score (Ln(K)) increased more slowly from *K* = 3–7 while Delta K supported three clusters (S1 Fig). The assignment plots associated with K = 3 showed no clear correspondence between geographical origin and cluster membership across sampling populations. On the individual level, admixture was evident in the majority of samples (Fig 4), confirming high levels of gene flow between Atlantic and Indian Ocean *G*. *galeus*.

### Demographic history of *Galeorhinus galeus*

Overall the parameters of neutrality for *G*. *galeus* presented by sampling location were indicative of population expansion rather contraction throughout the Southern Hemisphere and South Africa. Across the Southern Hemisphere, both South Africa and Argentina showed signatures of population expansion with statistically significant negative Tajma’s D and/or Fu’s values. This was corroborated by results of goodness-of-fit tests for the observed mismatch distributions, which were non-significant (*P* > 0.05) for all of the geographic sampling populations (Table 6), suggesting past population expansion. However, the expansion model was rejected for Chile as well as for the pooled Southern Hemisphere samples as indicated by the multimodal curve of mismatch distributions while a significant deviation from mutation-drift equilibrium (D = 0.338, *P* = 0.652) was also not evident. Since the haplotype genealogies depict three clades most likely linked to continental shelves, the analysis of demographic history was also presented by clade rather than by sampling location. Tests for neutrality indicated a departure from mutation-drift equilibrium for all three clades and the unimodal curves detected for the mismatch distribution was also indicative of populations having passed through a recent demographic expansion. Although the observed mismatch distribution was compared to two models of population development, the expansion and decline model versus the constant model, these observations were carefully interpreted as observed mismatch distributions may be a consequence of several demographic processes.

**Table 6:** Demographic analysis parameters for mtDNA *ND2* sequences of all sampling populations of *Galeorhinus galeus*.

Site/clade	Neutrality tests			
	D	F_S_	SSD	H_R_
**CHI**	0.338 (*P* = 0.652)	0.381 (*P* = 0.508)	0.064 (*P* = 0.320)	0.222 (*P* = 0.580)
**ARG**	-0.521 (*P* = 0.321)	-1.758 (*P* = 0.039)	0.028 (*P* = 0.240)	0.191 (*P* = 0.190)
**SA**	-1.946 (*P* = 0.003)	-5.109 (*P* = 0.000)	0.012 (*P* = 0.180)	0.571 (*P* = 0.680)
**AUS**	-1.088 (*P* = 0.200)	-0.264 (*P* = 0.169)	0.307 (*P* = 0.080)	0.358 (*P* = 0.310)
**NZ**	no polymorphism	no polymorphism	n.d.	n.d.
**SH all**	-1.030 (*P* = 0.100)	-2.343 (*P* = 0.050)	0.082 (*P* = 0.164)	0.268 (*P* = 0.352)
**RI**	-1.600 (*P* = 0.040)	-0.537 (*P* = 0.117)	0.004 (*P* = 0.600)	0.262 (*P* = 0.650)
**FB**	-1.237 (*P* = 0.125)	0.856 (*P* = 0.598)	0.045 (*P* = 0.260)	0.673 (*P* = 0.730)
**KL**	-1.451 (*P* = 0.069)	0.432 (*P* = 0.358)	0.015 (*P* = 0.240)	0.750 (*P* = 0.690)
**AB**	-1.048 (*P* = 0.148)	1.688 (*P* = 0.767)	0.102 (*P* = 0.090)	0.680 (*P* = 0.780)
**SB**	-1.149 (*P* = 0.163)	-0.537 (*P* = 0.020)	0.000 (*P* = 0.380)	0.503 (*P* = 0.750)
**PE**	no polymorphism	no polymorphism	n.d.	n.d.
**SA all**	-2.299 (*P* = 0.010)	-4.213 (*P* = 0.020)	0.094 (*P* = 0.173)	0.478 (*P* = 0.390)

Demographic indices: Neutrality test estimates Tajima’s test (D) and Fu’s test (F_S_), sum of squared distribution (SSD), Harpending’s raggedness index (H_R_), n.d. Not determined due to lack of polymorphism.

On a local scale, only the collection of Robben Island showed significant Tajima’s D value (D = -1.600, *P* = 0.040) reflecting an excess of rare polymorphisms and population expansion in the past (Table 6). Significant deviation was also observed overall populations (D = -2.299, *P* = 0.010). This was further supported by the non-significance for the sum of squares distribution (SSD) and relatively low levels of Harpending’s raggedness index obtained for all sampling populations. For the entire South African dataset, a process of expansion is suggested by the unimodal curve of mismatch distributions but was not statistically supported (F_S_ = -0.493, *P* = 0.490 and SSD = 0.050, *P* = 0.192). The latter observation of deviation from neutrality for the pooled South African dataset could well be an artefact of sampling in that the South African samples do not necesarrily represent a panmictic population assumed not to be affected by local, rapid demographic processes [65].

## Discussion

### Population genetics of Southern Hemisphere *Galeorhinus galeus*

Patterns of contemporary and historical gene flow were determined for *G*. *galeus* across the South Pacific, South Atlantic and the Indian Ocean. Both mitochondrial and nuclear data indicate that the species is highly divergent across the three ocean basins and the hypothesis of panmixia can be rejected based on statistical support. Since only a small number of individuals were assessed per sampling population, results are placed in context by comparing the overall genetic diversity estimates obtained for *G*. *galeus* in this study with other elasmobranch species exhibiting similar life history patterns. Overall, the results show relatively low genetic diversity for *G*. *galeus* across the Southern Hemisphere. The overall *ND2* haplotypic diversity (*h* = 0.626) is comparable to those reported for other commercially exploited shark species such as *Mustelus mustelus*, *Sphyrna lewini*, *Carcharhinus brachyurus and C*. *falciformis* [7,26,27,66]. Although lower haplotype and nucleotide diversities are expected for coastal sharks with smaller distribution ranges than for pelagic shark species with wider distribution ranges (*e*.*g*. *Prionace glauca* and *Carcharhinus falciformis*), recent studies have reported low levels of genetic diversity also for highly migratory pelagic species including *Pseudocarcharias kamoharai* [67] and *Carcharhinus longimanus* [68].

Based on mtDNA *ND2* haplotypes, this study confirms historical dispersal for *G*. *galeus* along continental shelves and over short geographic distances with CHI and ARG as well as NZ and AUS sharing a single haplotype. This is supported by pairwise Ф_ST_ values and haplotype genealogy showing association with geographical distance. These results are in agreement with previous findings for this species, suggesting a lack of historical gene flow across the large open expanses of the South Atlantic, South Pacific and South Indian oceans [33]. The study by Chabot and Allen., 2009 [33] also postulated that South America had only one historical population but placed uncertainty on the interpretation of results due to low sample sizes used *e*.*g*. one sample from Argentina pooled with 11 samples from Peru. Divergent lineages with geographic correspondence, as was seen with *G*. *galeus*, can result from two alternative scenarios; vicariance or lineage sorting [69]. Since it is difficult to confidently ascertain lineage sorting, two models of vicariance were considered; the closure of the Tethyan corridor (12 to 20 million years ago) [70] and the emergence of the Isthmus of Panama (3.5 million years ago) [71]. The closure of the Tethyan corridor occurred at a time when the African and Eurasian plates converged, resulting in the elimination of the warm coastal Tethyan corridor between the Atlantic and Indian oceans [72]. The network genealogies indicated three clades which correspond to a South American, African and an Australian-New Zealand lineage, with shallow divergence between the latter two lineages. This is in accordance with both the global and regional studies of tope shark based on the mtDNA control region [18,33] suggesting vicariance as a result of the emergence of the Isthmus of Panama rather than an ancient divergence due to closure of the Tethyan corridor. Demographic analysis based on the mtDNA data set also suggested and confirmed recent population expansions for all of the Southern Hemisphere collections except Chile. The study by Hernández et al., 2015 [18] reported a similar demographic event for Australian and New Zealand tope shark that is characterised by a long historical period of population expansion that most likely began before the last glacial Pleistocene (19,000 years before present). It is likely that after the rise of the Isthmus of Panama, and the subsequent warmer interglacial period, new habitats opened up and promoted population expansion in the Southern Hemisphere countries within Atlantic and Indian waters. This demographic pattern is also observed in other shark species such as *S*. *lewini* [26], *Carcharhinus limbatus* [36], *C*. *brachyurus* [27] and *C*. *leucas* [73], which showed dramatic population expansion trends during the Pleistocene. Despite the evidence for population expansion in many species, coordinated expansion events across populations are not expected to be observed unless shared environmental and historical factors obscured evidence of lineage specific adaptation, as seen in elasmobranchs inhabiting a similar environment across a small spatial scale [e.g. for *G*. *galeus* in southern Australia and New Zealand [18] and *P*. *glauca* in the Pacific Ocean [37]. This synchrony in population expansions supports the argument that current genetic variation may be the result of a major regional event over all populations. However, as one can not assume that samples were drawn from a panmictic population but most likely from locally adapted populations [66], this study does not necassarily have samples from the appropriate spatial and temporal scales to determine the environmental changes associated with the historical events that influenced population dynamics of *G*. *galeus* across the Southern Hemisphere.

The microsatellite data did not support contemporary gene flow between all geographic sampling sites implying that known biogeographic barriers in the Southern Hemisphere can hinder gene flow for *G*. *galeus* over smaller and larger spatial scales. For example, the Mid-Atlantic and Benguela barriers together with the presence of gyres and straits possibly restricts gene flow between sampling populations of Argentina and South Africa while the Tasman Sea and the Great Australian Bight (GAB) are likely barriers between Australian samples and that of New Zealand. It should be noted that a panmictic population of *G*. *galeus* was previously found to exist between Australia and New Zealand [18] and did not include samples west of the GAB barrier. The high connectivity observed between South African and Australian samples in the current study is in accordance with recent studies of highly migratory sharks such as white shark (*Carcharodon carcharias*) [74] and the tiger shark (*Galeocerdo cuvier*) [75] and highlights the high dispersal ability for this relatively smaller bodied shark. Hierarchical Bayesian clustering assignment further supported a connection of Indian and Atlantic Ocean *G*. *galeus* with migration around the tip of South Africa, most likely associated with the Agulhas leakage [67]. Previous tagging efforts across the Southern Hemisphere have shown that *G*. *galeus* exhibits extensive migratory patterns within the Indian and Pacific ocean basins [19,21]. On a local scale, McCord 2005 [21] showed that *G*. *galeus* aggregates during autumn (March to May) and spring (September to November) when water temperatures are slightly cooler. Across the Tasman Sea, Hernández et al., 2015 [19] showed that *G*. *galeus* migrates between New Zealand and southern Australia and that these migrations occur more often over time. Similar to aggregation patterns noted in South Africa, *G*. *galeus* tends to aggregate in large numbers during spring and summer within the South West Atlantic (SWA), in closed gulfs and bays of northern Patagonia, and are believed to be the primary nursery grounds for the species [76]. Also, Cuevas et al., 2014 [77] studied the diving behaviour of *G*. *galeus* in the main nursery ground for the SWA and showed that the species prefers cool temperate waters ranging from temperatures between 17°C and 19°C and exhibits a yo-yo oscillatary movement within the water column. The seasonal migratory patterns exhibited by the aforementioned studies seem to indicate that *G*. *galeus* habours nursery grounds within coastal bay areas and seasonally aggregates towards these sites. Further understanding these migratory patterns will play an important role in the development of informed fishing and regulatory policies, particularly regarding protection measures for critical habitats such as *G*. *galeus* nursery grounds.

### Local population connectivity and management implications

On a local scale, no inter-oceanic *ND2* divergence was observed across the Atlantic/ Indian Ocean boundary, illustrating high levels of connectivity across the South African coastline. A single genealogical clade was detected indicating historical admixture between the Indian and Atlantic Ocean or quite possibly incomplete lineage sorting due to recent co-ancestry. The overall low haplotypic diversity in combination with a single haplotype shared by most of the individuals is similar to what was found for *Mustelus mustelus* [7] and *Carcharodon carcharias* [74] assessed along the South African coastline. Given that the latter studies are based on different mitochondrial genes, it is possible that the low haplotype diversity observed here simply reflects the inherent properties of the mitochondrial ND2 gene or the relatively short gene region sequenced (599bp). However, the haplotype network for the Southern Hemisphere mirrored the haplotype geneology previously obtained for the control region [18,33] and recently a few studies have demonstrated strong intraspecific divergence based on the ND2 gene [78,79]. The presence of only a few private haplotypes could therefore well indicate the lack of localized haplogroups expected for a species that shows philopatric behavior of females [80]. Bigger sample sizes and movement studies could help to confirm the presence or absence of philopatry in South African *G*. *galeus*.

The microsatellite dataset, and pairwise F_ST_ and standardized G”_ST_ values in particular, confirmed low but significant levels of genetic differentiation amongst local populations. Very similar levels of heterogeneity in microsatellite allelic distributions has recently been reported for smaller coastal shark species over range-wide and more restricted distributions including *M*. *mustelus*, *M*. *henlei* and *Carcharhinus isodon* [7,81,82]. Strong intra-oceanic differentiation was evident amongst samples of the Indian Ocean (SB en PE), illustrating contemporary restriction to gene flow along the south-east coast of South Africa. The Bayesian cluster analysis showed high levels of admixed assignment across all sampling populations with Port Elizabeth as the only population showing a more distinct membership. These findings support the hypothesis that an additional barrier besides the Atlantic/Indian Ocean boundary might be at play [83] and that the fragmentation of the PE population could be as a result of the cold water pockets found at the thermal front in this region. Although it seems the Bayesian clustering analysis was unable to resolve population genetic structure on a local scale, we hypothesize that the genetic differentiation across the South African coastline are probably a result of a combination of habitat preference, thermal fronts that generate cold water pockets and upwelling currents. In a previous study including only one collection of Indian Ocean samples, the varying levels of genetic admixture found across the South African coastline for *G*. *galeus* were predicted to occur as a result of habitat preference [23] and could therefore also be linked to the bioregions found along the South African coastline. More recently, Maduna et al., 2017 [84] also implicated the Cape Agulhas boundary as the main barrier to gene flow in four coastal shark species including *G*. *galeus*. Most noteworthy in the aforementioned study is that the coalescent analysis of migration supported assymetric gene flow of *G*. *galeus* from the Indian to the Atlantic Ocean, concordant with the Atlantic Ocean–Indian Ocean connection of *G*. *galeus* via Agulhas leakage proposed in the current study.

The outcomes of this study could have immediate implications for the local and more global management of *Galeorhinus galeus*. On a Southern Hemisphere scale, all sampled populations comprise distinct genetic groups and therefore management units in fisheries terms. This implies that any form of replenishment in the Pacific, Atlantic and Indian oceans will have to be done locally, without any genetic input from geographically distant populations. For local samples of *G*. *galeus*, the genetic data suggests that there could be more than one contemporary barrier affecting gene flow along the South African coastline. Although this do not result in fully differentiated ‘stocks’ in the classic fisheries sense, local managers should recognise the existence of a highly admixed population along the south-west coast and possibly more discrete populations on the east coast. We therefore suggest that locally, *G*. *galeus* should be managed, not just on an ecosystems-based approach in line with the marine bioregions of South Africa, but it should be taken into account that since most of the fishery efforts are centered on the southwestern coast, *G*. *galeus* of Atlantic origin might be most vulnerable. Furthermore, differences exhibited in mitochondrial haplotypes and microsatellite genotypes, between these and other populations included from the Southern Hemisphere, could facilitate trade-monitoring efforts for internationally traded products such as fins and meat which are known to be exported from South Africa to other countries.

## Supporting information

S1 Table Regional and local sampling populations, collection site, sample numbers (N) and sampling year. (DOCX)

S1 Fig L (K) distributions using the “log probability of data” (Mean of LnP±1) approach prior to application of Evanno method (above) and Delta K analysis of the true number of clusters following the Evanno method (below) across the Southern Hemisphere (left) and across South African (right). (DOCX)

S2 Fig L (K) distributions using the “log probability of data” (Mean of LnP±1) approach prior to application of Evanno method (above) and Delta K analysis of the true number of clusters following the Evanno method (below) for the two main genetic clusters Group 1 (left) and Group 2 (right) identified using STRUCTURE. (DOCX)

S3 Fig Individual cluster membership of the Southern Hemisphere samples following hierarchical structure performed on the two main genetic clusters (Group 1 and Group 2) identified using STRUCTURE. (TIF)

S4 Fig Plots of the isolation-by-distance (IBD) analysis of the South African sampling populations showing regression linearized F_ST_ and geographic distance (R^2^ = 0.238, *P* = 0.1478). (DOCX)
