# Supplementary material for: Population genetics of Southern Hemisphere tope shark (Galeorhinus galeus): Intercontinental divergence and constrained gene flow at different geographical scales
Source: PLoS One. 2017 Sep 7;12(9):e0184481. doi: 10.1371/journal.pone.0184481 (PMC5589243; doi:10.1371/journal.pone.0184481)
Supplement: S1 Table — (DOCX) [file pone.0184481.s001.docx]

| **Sampling population** | **N** | **Coordinates** | **Sampling year** |
| --- | --- | --- | --- |
| Chile (CHI) | 22 | S 33 26.327 W 70 39.316 | 2006 |
| Argentina (ARG) | 10 | S 40 32.599 W 62 12 00 | 2012 |
| South Africa (SA) | 124 | - | 2009-2015 |
| Australia (AUS) | 9 | S 43 37.878 E 145 58.125 | 2013 |
| New Zealand (NZ) | 20 | S 46 34.313 E 166 53.747 | 2010 |
| Robben Island (RI) | 33 | S 33 48.611 E 18 22.495 | 2009-2012 |
| False Bay (FB) | 11 | S 34 11.645 E 18 34.376 | 2012 |
| Kleinmond (K) | 37 | S 34 20.484 E 19 02.128 | 2012 |
| Agulhas Bank (AB) | 10 | S 34 53.183 E 19 58.916 | 2012 |
| Struisbaai (SB) | 26 | S 34 43.982 E 20 05.013 | 2013 |
| Port Elizabeth (PE) | 10 | S 33 44.776 E 25 34.834 | 2015 |
