# Supplementary material for: Population genetics of Southern Hemisphere tope shark (Galeorhinus galeus): Intercontinental divergence and constrained gene flow at different geographical scales
Source: PLoS One. 2017 Sep 7;12(9):e0184481. doi: 10.1371/journal.pone.0184481 (PMC5589243; doi:10.1371/journal.pone.0184481)
Supplement: S1 Fig — (DOCX) [file pone.0184481.s002.docx]

S1 Figure. L (K) distributions using the “log probability of data” (Mean of LnP±1) approach prior to application of Evanno method (above) and Delta K analysis of the true number of clusters following the Evanno method (below) across the Southern Hemisphere (left) and across South African (right)**.**
