# Supplementary material for: Population genetics of Southern Hemisphere tope shark (Galeorhinus galeus): Intercontinental divergence and constrained gene flow at different geographical scales
Source: PLoS One. 2017 Sep 7;12(9):e0184481. doi: 10.1371/journal.pone.0184481 (PMC5589243; doi:10.1371/journal.pone.0184481)
Supplement: S2 Fig — (DOCX) [file pone.0184481.s003.docx]

S2 Figure. L (K) distributions using the “log probability of data” (Mean of LnP±1) approach prior to application of Evanno method (above) and Delta K analysis of the true number of clusters following the Evanno method (below) for the two main genetic clusters Group 1 (left) and Group 2 (right) identified using STRUCTURE.
