# Supplementary figures and images for: Population genetics of Southern Hemisphere tope shark (Galeorhinus galeus): Intercontinental divergence and constrained gene flow at different geographical scales
Source: PLoS One. 2017 Sep 7;12(9):e0184481. doi: 10.1371/journal.pone.0184481 (PMC5589243; doi:10.1371/journal.pone.0184481)

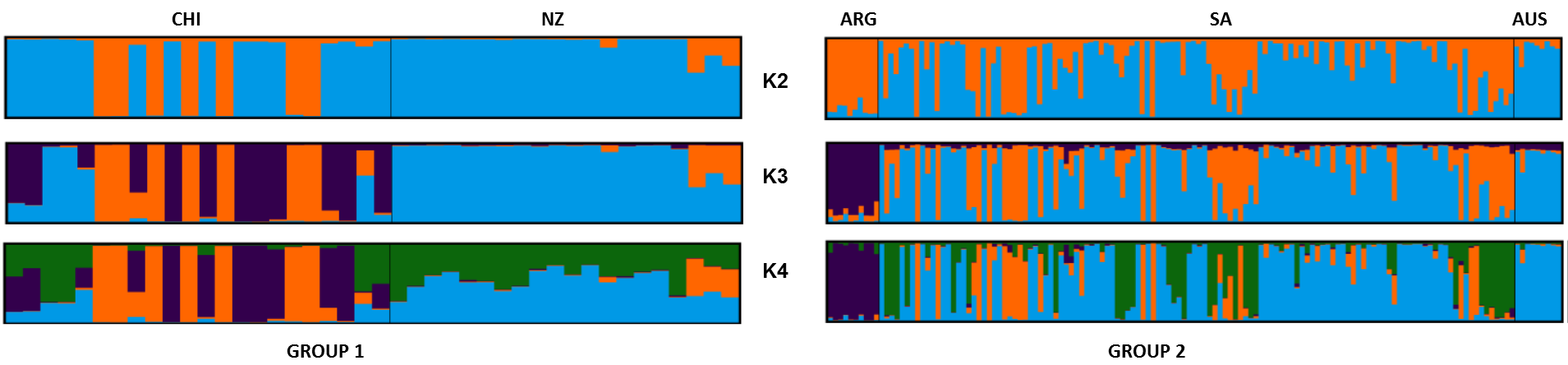

Supplement: S3 Fig — (TIF) [file pone.0184481.s004.tif]
