# Supplementary material for: Population genetics of Southern Hemisphere tope shark (Galeorhinus galeus): Intercontinental divergence and constrained gene flow at different geographical scales
Source: PLoS One. 2017 Sep 7;12(9):e0184481. doi: 10.1371/journal.pone.0184481 (PMC5589243; doi:10.1371/journal.pone.0184481)

S3 Figure. Plots of the isolation-by-distance (IBD) analysis of the South African sampling populations showing regression linearized F_ST_ and geographic distance (R^2^ = 0.238, *P* = 0.1478).


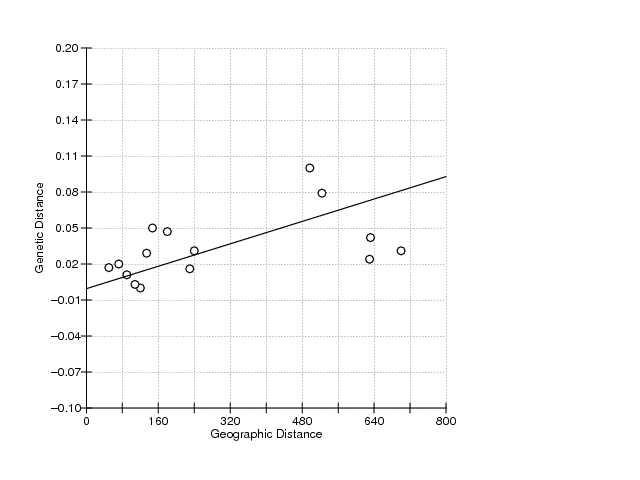

Supplement: S4 Fig — (DOCX) [file pone.0184481.s005.docx]
